# Thyroid Hormone Indices in Computer Workers with Emphasis on the Role of Zinc Supplementation

**DOI:** 10.3889/oamjms.2016.041

**Published:** 2016-03-15

**Authors:** Ahmed Ibrahim Amin, Noha Mohamed Hegazy, Khadiga Salah Ibrahim, Heba Mahdy-Abdallah, Hamdy A. A. Hammouda, Eman Essam Shaban

**Affiliations:** 1 *Chemistry Department, Faculty of Science, Cairo University, Egypt*; 2 *Environmental & Occupational Medicine Department, Environmental Research Division, National Research Centre, Egypt*

**Keywords:** Electromagnetic field, computer, Zinc, thyroid hormones, TSH, FT3, FT4

## Abstract

**AIM:**

This study aimed to investigate the effects of computer monitor-emitted radiation on thyroid hormones and the possible protective role of zinc supplementation.

**MATERIAL AND METHODS:**

The study included three groups. The first group (group B) consisted of 42 computer workers. This group was given Zinc supplementation in the form of one tablet daily for eight weeks. The second group (group A) comprised the same 42 computer workers after zinc supplementation. A group of 63 subjects whose job does not entail computer use was recruited as a control Group (Group C). All participants filled a questionnaire including detailed medical and occupational histories. They were subjected to full clinical examination. Thyroid stimulating hormone (TSH), free triiodothyronine (FT3), free thyroxine (FT4) and zinc levels were measured in all participants.

**RESULTS:**

TSH, FT3, FT4 and zinc concentrations were decreased significantly in group B relative to group C. In group A, all tested parameters were improved when compared with group B. The obtained results revealed that radiation emitted from computers led to changes in TSH and thyroid hormones (FT3 and FT4) in the workers.

**CONCLUSION:**

Improvement after supplementation suggests that zinc can ameliorate hazards of such radiation on thyroid hormone indices.

## Introduction

The problem of the influence of Electromagnetic Fields (EMFs) on biological systems has a long history. Now this problem attracts more public interest owing to increased electromagnetic pollution of the environment [1, 2].

Both industrial and domestic appliances constantly expose people to electric (E) and magnetic (M) fields. The EMFs are used not only for technological applications such as computer, power lines, mobile phones, house hold appliances, but are also widely applied for medical diagnosis and therapeutic purposes [3].

Some epidemiologic studies had shown associations between exposure to extremely low frequency - electromagnetic fields (ELF–EMF) and increased health risk in individuals working or living in environments exposing them to those fields. This was evidenced by increased incidence of certain types of cancer [4] and miscarriage [5], however, others have not shown such a link [6]. The human body functions are regulated by electric currents so it is expected that human physiological processes may be affected by exposure to EMF of sufficient strength [7]. Scientific interest has emerged about the mechanisms of the interaction between ELF-EMFs and living organisms. Animal studies had shown that ELF-EMFs may interfere with chemical reactions involving free radical production mechanisms [8, 9]. It has been reported that personal computer monitor use is associated with the free radical process [10].

Concerning the endocrine system, the sensitivity of pineal gland, pituitary gland, adrenal gland and thyroid gland as well as of the pancreas, testicles and ovaries to EMFs had been investigated [11]. One of the most exposed and vital organs are the thyroid gland. It is a target for different types of electromagnetic radiation [12]. It is stated that even minimal change in thyroid hormone levels circulating in the blood is sufficient to alter the brain functions of individuals [13]. However, only few published papers studied the effect of ELF–EMF emitted by computer on human’s thyroid gland hormones [14].

Owing to the scarcity of data on the effects of ELF emitted by computer -induced electromagnetic fields on the TSH and thyroid hormones in humans, the current work aimed to assess the potential alterations of thyroid hormones in computer workers and the possible protective role of zinc supplementation.

## Subjects and Methods

The study was conducted in the internet unit of the National Research Centre. It included forty-two computer workers (group B). They were subdivided into 19 males and 23 females. They worked daily for a mean duration of 8 ± 1.2 hrs for 5 days weekly. The mean duration of exposure to computer was (14.3 ± 8) years. Zn supplementation was given to the computer workers who were willing to continue the study (group A) in the form of Octazinc tablets (containing zinc sulphate 120 mg equivalent to 25mg zinc). The administered dose was one tablet/day for 8 weeks. A comparable group of 63 persons matched for age and socioeconomic status were included as a control group (group C). It consisted of 33 males and 30 females. Their work does not entail computer use. But they use computers for purposes other than work. They use computer for a mean duration of (4 ± 0.8) hours/day for 5 days weekly. They are exposed to electromagnetic radiation for a lower duration (7.2 ± 2.0) years. Subjects with histories of liver diseases, thyroid gland abnormalities, exposure to toxic substances and shift work were excluded from the sample. Written consents were taken from all participants. Approval of the Ethical Committee of Medical research of the National Research Centre was obtained in advance.

### 

#### Environmental Measures

Working with computers is carried out in closed rooms with appropriate humidity, ventilation and temperature. EMFs were measured at the work place using the Digital electrostress analyzer device ME 3030B (frequency range 5 Hz to 100 kHz, EF range: 1 V/m – 1999 V/m and MF range: 1 nT-1999 nT or 0.01 mG-19.99 mG). The device was purchased from Gigahertz Solutions Company, Germany.

#### Biochemical Parameters

A sample of about 5 ml of venous blood was obtained and placed in heparinized test tubes. Blood samples were taken from worker before zinc supplementation at 12 p.m. (2 h after the start of work) and from control subject. After eight weeks of Zn intake, another sample was obtained from computer workers. Blood was centrifuged (3000 rpm; 10 min, 4°C) then serum was separated and stored in the refrigerator until analyzed. Zinc concentration was measured by spectrophotometric method [15]. The level of FT3 [16], FT4 [17], and TSH [18] were measured by ELISA kits from Dia Metra, Italy.

#### Statistical analysis

The obtained results were expressed as mean ± SD. The statistical difference between various groups was analyzed by the one-way ANOVA and the significance was set at p≤0.05. Relation between variables was studied using Pearson Correlation. Figures were illustrated using excel program.

## Results

Electromagnetic radiation emitted from computer monitor plus control processing unit (CPU) was measured when computers are turned on. Measurements were obtained at frequency 50 Hz. The mean value of magnetic field was 0.167 ± 0.042 and 0.151 ± 0.039 μT at distances 30 and 50 cm respectively. The mean value of electric field was 386 ± 58 and 253 ± 51 m/v at distances 30 and 50 cm respectively.

The mean age of the computer workers was 38.1 ± 9.6 years. The mean age of the control group was 37.95 ± 8.4 years. There was no statistical difference between the two groups concerning age.

As shown in Table 1 TSH, FT3 and FT4 levels were significantly lower in group B female when compared with group C female. There was no significant difference in group B male when compared with group C male. And this decrease in TSH level in group B female was corrected after supplementation.

**Table 1:** TSH, FT3 and FT4 levels among males and females of studied groups

	Group	Gender	Mean ± SD	LSD	ANOVA	

					F-ratio	P-value
TSH (mlu/L)	Group C (No=63)	M (33)	2.376 ± 0.50	----	2.740	< 0.05

		F (30)	2.502 ± 0.55	(BM, BF)		

	Group B (No=42)	M (19)	2.096 ± 0.44	(CF, AF)		

		F (23)	2.068 ± 0.32	(CF, AF)		

	Group A	M	2.135 ± 0.29	(AF)		

		F	2.551 ± 0.37	(BM, BF, AM)		

FT3 (ng/dl)	Group C (No=63)	M (33)	2.14 ± 0.497	----	1.718	NS

		F (30)	2.29 ± 0.650	(BF)		

	Group B (No=42)	M (19)	1.94 ± 0.253	----		

		F (23)	1.74 ± 0.463	(CF, AF)		

	Group A	M	2.07 ± 0.517	----		

		F	2.20 ± 0.05	(BF)		

FT4 (ng/dl)	Group C (No=63)	M (33)	1.09 ± 0.112	(BF)	2.680	< 0.05

		F (30)	1.10 ± 0.088	(BF)		

	Group B (No=42)	M (19)	1.05 ± 0.156	----		

		F (23)	0.96 ± 0.168	(CM, CF, AF)		

	Group A	M	1.03 ± 0.126	(AF)		

		F	1.14 ± 0.085	(BF, AM)		

M: male, F: Female. CM: Control group male, CF: Control group female, BM: before supplement male, BF: before supplement female, AM: After supplement male, and AF: After supplement female, NS: non-significant.

Table 2 showed a significant difference in zinc concentration between group B female and group C female. There was no significant difference between female and male in group B. Zn level was improved in females of group B.

**Table 2:** Comparison of zinc concentration between the three studied groups according to gender

Group	Gender	Mean ± SD	LSD
Group C (No = 63)	Male (33)	130.4 ± 22.4	----

	Female (30)	148.1 ± 23.5	BF

Group B (No = 42)	Male (19)	130.3 ± 18.4	----

	Female (23)	119.9 ± 19.02	(CF, AM, AF)

Group A	Male	139.3 ± 15.7	BF

	female	137.8 ± 21.7	BF

ANOVA	F-ratio	2.371	

	P-value	= 0.05	

CM: group C male, CF: group C female, BM: group B male, BF: group B female, AM: group A male, AF: group A female.

**Figure 1:**
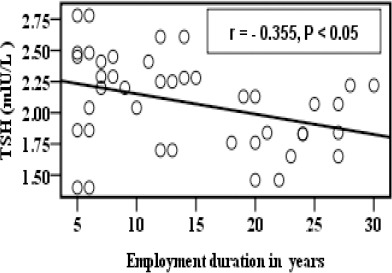
*Correlation between zinc concentration and employment duration in years*.

**Figure 2:**
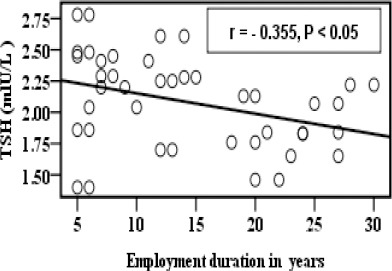
*Correlation between TSH and employment duration in years in group B*.

Figure 1 showed significant negative correlation between employment duration in years and zinc concentration before supplementation. Employment duration in years was found to be negatively correlated with TSH in the group B as shown in Figure 2.

**Figure 3:**
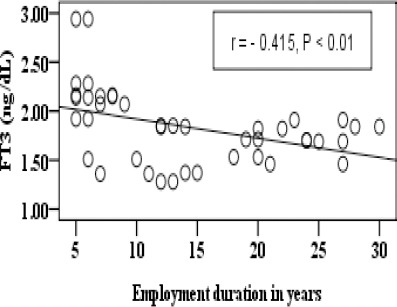
*Correlation between FT3 and employment duration in years in group B*.

There was a significant negative correlation between employment duration of exposure in years and FT3 in group B as illustrated in Figure 3.

**Figure 4:**
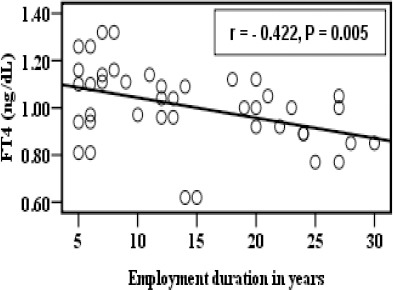
*Correlation between FT4 and employment duration in years in the group B*.

There is a significant negative correlation between employment duration of exposure in years and FT4 in group B as shown in Figure 4.

## Discussion

Computer usage is growing in industrialized societies. The computer video display unit is the most critical part in respect of electromagnetic radiation emission especially those of the cathode ray tube type. Electromagnetic hazards are considered one of the most dangerous types of pollution which affects the functions of body cells [19].

The basic electronics of Video display units (VDUs) produce electro-magnetic fields of wide frequency range – from several hertz up to half of megahertz. A computer user is exposed to visible light, ultraviolet light, and ELF- EMF. The ELF- EMF emitted from VDUs comes from the power supply, transformers, and the vertical deflection coils. Weak signals at higher radio frequencies (RF) come from the VDUs’ interior electronic circuit. Very low-energy X-rays are produced inside the CRT, but the glass screen is thick enough to completely absorb them before they escape from the VDU [20]. Preliminary experiments showed that radiation from a monitor can produce potentially hazardous biological effects [10].

The examined computer workers in this study are exposed to very low magnetic field compared to the accepted limits for occupational exposure by the National Radiological Protection Board which is 500 μT. However, our results showed significant decrease in serum zinc levels in computer workers (group B) compared with the controls (group C). That finding comes in consistent with Ozturk et al [21] who reported that serum zinc levels were decreased significantly in rats exposed to EMF. Therefore reduced zinc levels may contribute to EMF–induced oxidant stress and explain its harmful effects.

Serum zinc levels significantly increased in computer workers after Zn intake (group A). This agreed with results obtained by several studies [21, 22]. The Zn level was much lower in female computer workers than male workers of group B. This gender difference suggests that females might be more prone to hazardous effects of EMF. Further studies on a larger sample are needed to confirm this finding. Zn intake markedly improved the level in females to reach (137.8 ± 21.7) compared to (119.9 ± 19.02) prior to supplementation.

Previous experimental studies demonstrated a variation in thyroid activity in rats after different durations of exposure to EMFs, as measured by serum levels of thyroid hormones or judged by the thyroid morphological features [14, 23, 24]. However, there are only few published papers that report the effect of ELF–EMF emitted by computer on human’s thyroid gland hormones [25].

Actually thyroid hormones in their free forms (Free -T3 and Free -T4), act more efficiently rather than bound forms (bound to plasma proteins). Therefore concentrations of Free-T3 and Free-T4 are better criteria to assess the activity of thyroid hormones. In this study, it was observed that thyroid functions was affected in computer workers as detected by lowered levels of (TSH, FT3, FT4) in group (B).

In addition the results revealed that there was a negative correlation between TSH, FT3, and FT4 and employment duration of exposure in years as shown in Figure 2, 3 and 4.

Our results are consistent with those of Zagorskaya et al [23]. Those authors found lowered concentration of thyroid hormones after 2 months following a single exposure of rats to 20mT ELF–EMF. Similarly, long-term exposure to ELF–EMF (50–500 μT) for 3 months decreased the serum TSH and tri-iodothyronine-thyroxin (T3–T4) levels in male rats [26]. Furthermore, previous investigations of ELF–EMF effect on thyroid gland showed that rats exposed to ELF–EMF fields demonstrated altered thyroid gland activity [27]. Also, Matavulj et al [28] found that thyroid gland showed increased activity after 2 months of ELF-EMF exposure and decreased activity after 5 and 6 months, as measured by histological and stereological parameters. They concluded that the type of alteration depends on the duration of exposure and it is reflected as either increased or decreased activity of the thyroid.

Moreover, De Seze et al [29] demonstrated a 21% decrease in TSH among male subjects chronically exposed to GSM cell phone fields for two hours per day, five days per week for one month. Additionally, Koyu et al [14] investigated the effect of 900 MHz GSM-like frequency EMF on serum TSH, T_3_ and T_4_ hormones levels in rats. The study revealed a lowered TSH and thyroid hormones. That decrease is possibly the result of tissue heating and is generally similar to nonspecific stress responses caused by EMF exposure in rats. Also, found that high frequency exposure elevated T4 and decreased T3 after 50 days exposure period [30]. Hosseini et al [31] found lowered levels of FT3 and FT4 in male rabbit after exposure to 10 Hz EMF and suggested that this lowering in FT3 and FT4 may be due to a decrease in TSH level.

Previous studies investigated the influence of 50Hz EMF (50–500 μT) for 2–6 months on 1 – day old male rats [25, 27, 32]. Results of those studies demonstrated the effect of EMF exposure on thyroid follicular epithelium, follicular colloid content, inter follicular connective tissue and mast cells. The resultant changes showed decreased thyroid activity after three, five and six months of exposure and increased thyroid activity after two months.

Several lines of evidence indicate that the morphological changes following electromagnetic field exposure are accompanied by some endocrine changes. It has been demonstrated that ELF-EMF evokes morphological changes in rat’s thyroid gland, affects its endocrine function and decreases plasma T4 and T3 concentrations [25, 27].

Zinc is a necessary trace element for the catalytic activity of several enzymes involved in the metabolism of hormones. Zinc effects on thyroid hormones are complex and include both synthesis and mechanism of action. Zinc with cysteine residues is present in thyroid transcription factors that are necessary for modulation of gene expression [33].

The influence of zinc on thyroid hormone levels and the thyroid gland generally is still unclear; although, preliminary evidence suggests that such nutrient has an important role [34]. In animal experiments, zinc deficiency, although having no impact on T4 concentrations, caused approximately thirty percent decline in levels of serum T3 and FT4. The activity of type I 5’-deiodinase was also decreased in zinc-deficient animals [35, 36]. Inhibition of conversion of T4 to T3 was similarly detected in an independent animal experiment [37]. They demonstrated a decrease in the serum T3 level of zinc-deficient rats and they suggested that zinc was as important as selenium or iodine for thyroid hormone homeostasis.

In humans, supplementation with zinc restored thyroid function to normal in disabled hypothyroid patients under anticonvulsant therapy. In a study nine of thirteen subjects with low free T3 and normal T4 had mild to moderate zinc deficiency. Following oral intake of zinc sulfate (4-10 mg/kg body weight for 12 months), levels of serum FT3 and T3 returned to normal, serum FT4 decreased, and the TRH-induced TSH reaction became normal [38].

Maxwell and Volpe [39] found that, although metabolic rates and thyroid hormone levels of two zinc-deficient subjects were comparable, zinc supplementation (26.4 mg/day as zinc gluconate) increased both free and total T3 and T4 levels in one subject, and only total T3 increased in the other subject at four months, however resting metabolic rate was increased in both. On the other hand some other researchers showed increase in serum TSH, FT3 and FT4 concentrations [40, 41]. Selmaoui et al [42] reported that there was insignificant difference in T3 and T4 between non-exposed males and men exposed to continuous and intermittent 50 Hz magnetic field of ten Tesla overnight. Additionally, an earlier study conducted by Lafreniere and Persinger [43] on ELF-EMF influence on thyroid gland showed absence of changes in T3 and T4 concentrations and number of thyroid follicles in rats exposed to 0.5 Hz EMF prenatally and/or in adults.

In conclusion, EMFs may have deleterious effects on thyroid gland activity. It is advised to include thyroid function tests in periodic medical examination of computer workers. Zinc supplementation may ameliorate the thyroid gland activity in such workers. Moreover, further investigations are required to clarify the degree of TSH and thyroid hormones alteration by electromagnetic field emitted from computers and the relation between zinc supplementation and thyroid hormones. Studies on a larger scale are needed to explain gender difference.
